# Participating in a Nutrition-Sensitive Agriculture Intervention Is Not Associated with Less Maternal Time for Care in a Rural Ghanaian District

**DOI:** 10.1093/cdn/nzac145

**Published:** 2022-09-29

**Authors:** Yvonne E Goh, Grace S Marquis, Esi K Colecraft, Richmond Aryeetey

**Affiliations:** School of Human Nutrition, McGill University, Sainte-Anne-de-Bellevue, Quebec, Canada; School of Human Nutrition, McGill University, Sainte-Anne-de-Bellevue, Quebec, Canada; Department of Nutrition and Food Science, University of Ghana, Legon, Ghana; School of Public Health, University of Ghana, Legon, Ghana

**Keywords:** child care, time allocation, women in rural settings, agriculture, Ghana

## Abstract

**Background:**

Nutrition-sensitive agriculture (NSA) interventions may increase farm-related work for mothers, with consequences for child nutrition. The Nutrition Links (NL) intervention provided mothers with poultry, gardening inputs, technical support, and education to improve livelihoods and child nutrition outcomes in rural Ghana.

**Objectives:**

Our objective was to compare time allocated to child care by a cross-section of mothers in the intervention group of the NL intervention with the control group (NCT01985243).

**Methods:**

A cross-section of NL mother-child pairs was included in a time allocation substudy [intervention (NL-I) *n *= 74 and control (NL-C) *n *= 69]. In-home observations of the mother-child pair were conducted for 1 min, every 5 min, for 6 h. Observations were categorized into 4 nonoverlapping binary variables as follows: *1*) maternal direct care, *2*) maternal supervisory care, *3*) allocare, and *4*) no direct supervision. Allocare was defined as care by another person in the presence or absence of the mother. Any care was defined as the observation of maternal direct care, maternal supervisory care, or allocare. Generalized linear mixed models with binomial data distribution were used to compare the child care categories by group, adjusting for known covariates.

**Results:**

Maternal direct care (OR = 1.07; 95% CI: 0.89, 1.28) and any care (OR = 1.56; 95% CI: 0.91, 2.67) did not differ by intervention group. However, there was a higher odds of allocare (OR = 1.36; 95% CI: 1.04, 1.79) in NL-I than in NL-C women.

**Conclusions:**

Maternal participation in an NSA intervention was not associated with a decrease in time spent directly on child care but was associated with an increase in care from other household and community members.

The clinicaltrials.gov number provided is for the main NL intervention and not this current substudy.

## Introduction

Time use is a mediator in the pathway between women's participation in agriculture and their children's nutrition (1). Compared with men, women have higher demands on their time in many settings, primarily because of defined gender roles (2–4). Although there is limited research on the time use of rural women, the available data suggest that women in rural settings are time-poor (5). A recent study that used accelerometers to track rural household activity in Ghana and India showed that women spent >60% of their time doing domestic and economic activities whereas men used 54% for these same activities (6). There appears to be a higher demand on women's time for domestic and economic activities, compared with men.

Nutrition-sensitive agriculture (NSA) interventions leverage women's participation in agriculture to improve livelihoods, food security, and nutrition outcomes in rural settings disproportionately affected by poverty and malnutrition (7). A review showed positive effects of nutrition-sensitive agriculture on dietary diversity, micronutrient status, hemoglobin concentrations, and diarrheal episodes in children across different settings in Asia and Africa (8). In Ghana, women's participation in nutrition-sensitive interventions was associated with improved dietary diversity, animal source food intake, and child growth outcomes (9, 10). However, NSA interventions can come with additional demands on mothers' time, which is already highly tasked; in these interventions, women were expected to be active participants for successful outcomes.

It has been hypothesized that the additional time burdens associated with interventions could have adverse consequences on child care and nutrition (11). Therefore, measuring rural maternal time use represents an opportunity to improve the understanding of the linkage between NSA and nutrition outcomes. Further, understanding the time use of women in rural settings will guide the development of appropriate strategies to prevent unintended detrimental consequences for children of maternal participation in NSA interventions. The current cross-sectional study assessed how participation in an NSA intervention affected mothers' time allocation to child care.

## Methods

### Study area

The study site, Upper Manya Krobo (UMK), is 1 of the 21 districts in the Eastern region of Ghana (12). This is a mostly rural agricultural district and has a total of 198 communities within 6 administrative subdistricts. Agricultural activities depend almost exclusively on 2 rainy seasons: early April to August, and September to October. Crop farming is the main livelihood in the majority of the communities. Farming is, however, at the subsistence level with limited use of mechanized agriculture technologies. The main food crops grown in UMK are cassava and maize. However, cowpea, mango, and other fruits and vegetables are also cultivated. The second most important livelihood in the district is trading. The district is a major commercial center for agricultural produce in the Eastern region, due to the presence of 3 large markets. Communities along the Volta Lake depend mainly on fishing as a source of livelihood. Basic infrastructure is generally inadequate. The road network is particularly poor, making transportation of people and market goods a major challenge. Access to potable water and electricity is limited to the more urban communities. The district is served by a hospital, maternal and child health clinics, and community-based health planning and services compounds.

### Nutrition Links intervention

The Nutrition Links (NL) project commenced in 2013 as a partnership between McGill University, World Vision, the University of Ghana, and local nongovernmental (Heifer Ghana and Farm Radio International), governmental (Ghana Health Service, District Office of Agriculture, and National Commission for Civic Education), and private (Upper Manya Krobo Rural Bank) institutions. The design, setting, randomization, and primary outcomes of the NL intervention have been previously described (10). Briefly, it included a series of institutional and community-based activities including an integrated agriculture and nutrition education trial that was carried out sequentially in two 12-mo phases, ∼1 y apart, and involving mothers of infants and young children.

This substudy involved mothers who were participating in the second phase of the trial. The first phase of infants and young children were aged 9.4 ± 3.9 mo, whereas the second were slightly older at 12.4 ± 6.3 mo. The intervention provided each woman in the second phase with *1*) technical support and transfer of poultry husbandry (30 point-of-lay Swiss Brown chickens) and horticultural inputs (seeds; 5–10 kg sweet potato vines; tomato and green leafy vegetable seedlings) for home gardening; *2*) weekly child nutrition and psychosocial stimulation education; and *3*) community-wide health-related education (food demonstrations, mother-to-mother support groups on infant and young child feeding, and gender and diversity training). Mothers in the intervention also received continuous technical support with poultry farming and home gardens.

### Study participants

**FIGURE 1:**
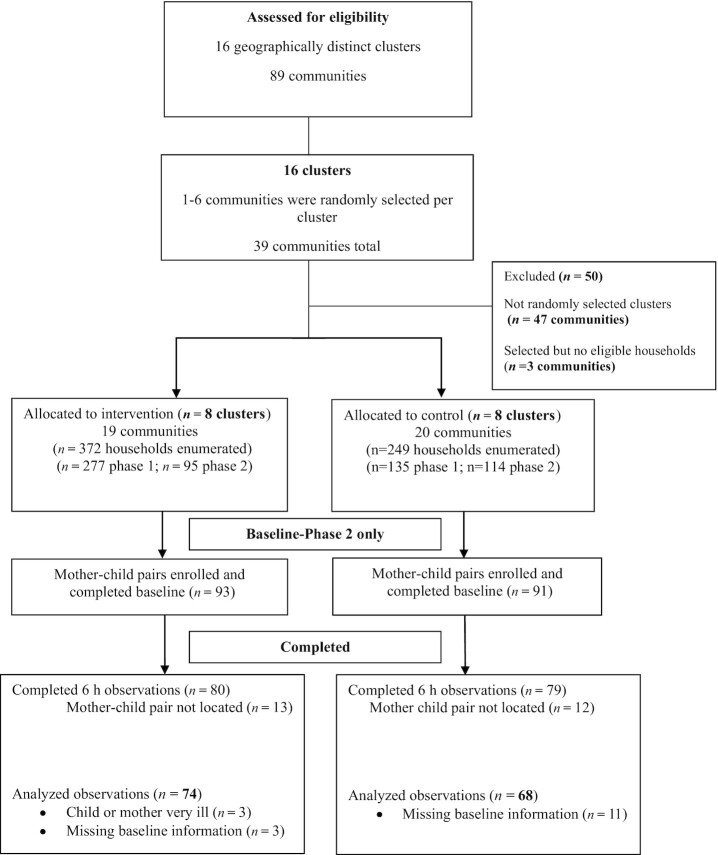
Participant flow through the study.

The study participants were a cross-sectional sample of the NL trial phase 2 participants. The sampling procedure for the main NL intervention has been previously described (10). A census was first conducted in 3 subdistricts of the UMK district. A total of 89 communities that were organized into 16 clusters were assessed for eligibility for the NL intervention. To ensure the selection of a minimum of 14 households with infants or young children per cluster for participation in NL intervention activities, a total of 39 communities were selected in each of the 16 clusters. Eight clusters (19 communities) were allocated to the intervention group (NL-I), and 8 clusters (20 communities) were allocated to the control group (NL-C). Of the eligible households, 93 intervention and 91 control group mother-child pairs completed the baseline survey for the second phase and were eligible to participate in this present study. The flow of participants through the study is shown in **Figure 1**.

### Data collection procedures

The 6-h direct observations of mother-child pairs were carried out between October and December 2016. The day of the week for the observations was randomly assigned to communities and excluded weekends. Field workers carried out an initial visit to inform mothers about the study and obtain informed consent. The mothers were then given information about the day of the visit for their respective communities and also informed to expect field assistants on that day of any of the coming weeks. Research assistants were standardized in their observations through a pretesting exercise. The in-home observations were carried out using focal person sampling (mother-child pair) involving 1-min sampling at 5-min intervals for a total of 6 h (13). Two research assistants were assigned to each mother-child pair; one observed the mother and the other the child with the aid of a hand-held watch. Each observation lasted 1 min; the remaining 4 min were used for recording observations. Every 50 min of observation was followed by 10 min of rest. The research assistants recorded observations on structured paper templates (**Supplemental Appendices A** and **B**). The research assistant observing the mother recorded all activities during the 1-min window, including whether the mother could see or hear her child, the location of the activity, and the persons present during the activity. Research assistants followed the mother everywhere she went (e.g., farm, market, riverside, clinic) to ensure that all activities were captured. Similar information was collected on the child, with critical attention paid to who was providing care to the child. This resulted in 2 sets of data: *1*) maternal observations, and *2*) child observations. The observations in both datasets were identical unless the mother and child were separated. Observations of the mother's hygiene practice were carried out for 4 key activities (meal preparation, mother eating, feeding the child, and cleaning the child after defecation) when they happened during the 1-min observation window. Whenever one of these activities was recorded, field assistants noted whether the mother washed her hands with just water, soap and water, or not at all. The hygiene score was calculated by counting the number of times the mother washed her hands with soap and water before meal preparation, eating food, and feeding the child. The number of times the mother washed her hands with soap and water after she attended to the child after defecation was also counted. The total number of times handwashing with soap was observed with the 4 activities was then divided by the total number of times these activities were observed in the 6-h observation period.

The Home Observation for Measurement of the Environment (HOME)—a tool associated with mental development—was used to assess the psychosocial stimulation of the child during the 6-h observation period (14, 15). This tool, adapted for use in low- and middle-income countries, has 45 simple binary response questions about the amount and quality of interactions in the home and the presence of learning and play materials available to the child. As recommended, the HOME was not assessed if the mother and child were not together for ≥45 consecutive minutes. The HOME score was the sum of positive responses out of the total of the 45-item questionnaire.

In addition to the direct observation data, a wide range of household-, maternal-, and child-specific information was available through the NL baseline survey. Relevant baseline data for this substudy included household (ownership of assets and household size), maternal (depressive symptoms and anthropometry), and child (diet intake and anthropometry) information.

The household asset index was calculated based on binary questions about the ownership of 13 household assets: floor material, wall material, cooking fuel, electricity, and ownership of a telephone, radio, television, video player, DVD/CD player, refrigerator, sewing machine, motorcycle, and car. The first component of the principal component analysis was then used as the wealth index (10). The 20-item Self Reporting Questionnaire (SRQ-20) was used to measure depressive symptoms in mothers (16). It included questions with binary responses on feelings of worthlessness, fatigue, difficulty concentrating, depressive moods, and other mental depressive symptoms that fall under “common mental disorders” (17). A depressive symptoms score was calculated as the sum of positive responses to the SRQ-20 questions (range 0–20). Validation studies of the SRQ-20 in low- and middle-income countries have demonstrated the scale's internal consistency (Cronbach α = 0.84) and have suggested a cut-off point of 5–6 out of 20 to provide the best balance between specificity and sensitivity (18, 19).

The child's dietary diversity score was assessed with a binary scale list-based FFQ, which was adapted for the local context. The answers to the food frequency questions were recategorized into 7 food groups (grains, roots, and tubers; legumes and nuts; dairy products; flesh food; eggs; vitamin A–rich foods and vegetables; and other fruits and vegetables). The percentage of children who met the WHO's recommended cut-off for minimum dietary diversity of 4 out of the 7 food groups was then calculated (20). Weight and height measurements for both the mother and child were taken in duplicate to the nearest 0.1 kg and 0.1 cm, respectively, using digital scales (Tanita Corp) and stadiometers (Shorr Productions), using recommended WHO standards (21).

### Data analysis

#### Child care outcome measures

Child care was coded using the data from both the maternal and child observations. The coding reflected whether the child received care at any observation time point, and who provided the care, irrespective of any other activity happening at the same time. This allowed for a maximum of 61 one-minute care observations for each child. Five variables were created to describe child care: *1*) maternal direct care (child care by the mother only), *2*) maternal supervisory care (mother is not providing direct care but can see or hear the child), *3*) allocare (child care by another person in the presence or absence of the mother), *4*) any child care (maternal direct care, maternal supervisory care, or allocare), and *5*) no supervision (child is left unattended to). Each child care variable was coded into a binomial variable (present/not present at each observation event), and the prevalence of each category was estimated.

#### Covariates

The covariates used in our analysis were child age, maternal age, maternal education, working status, maternal BMI, depressive symptoms score, household wealth index, and household size. These were selected based on literature and factors that could potentially influence the amount of care a child received in a household.

#### Analysis approach

The binomial child care outcomes [*1*) maternal direct care, *2*) maternal supervisory care, *3*) allocare, *4*) any child care, and *5*) no supervision] were used in separate generalized linear mixed models to compare the differences in child care by intervention group. Specifically, SAS PROC GLIMMIX (SAS Institute Inc) with the logit function was used while accounting for the random effect of cluster and predictor variables (child age, maternal age, maternal education, working status, maternal BMI, depressive symptoms, household wealth index, and household size). For predictor categorical variables with 3 levels (child age, maternal age, and education), the Dunnett test was used to adjust the *P* values for multiple comparisons (22).

### Ethical approval

The institutional review boards of McGill University and the Noguchi Memorial Institute for Medical Research at the University of Ghana provided ethics approval for the trial. Informed consent was obtained from all mothers in the study. The trial was registered at clinicaltrials.gov (NCT01985243).

## Results

Time-use data were collected for 159 mother-child pairs; however, only data for 142 mother-child pairs were included in the analysis (74 NL-I and 68 NL-C pairs) due to missing background information or incomplete data because of severe illness in the mother or child that required hospitalization (**Figure 1**). There were few differences between trial arms (**Table 1**). More mothers were Krobo (local ethnic group), and fewer were married or belonged to households with fewer assets in the NL-I compared with the NL-C group. On average, the children's age at the time of observation was ∼21 ± 6.4 mo.

**TABLE 1:** Baseline characteristics of study participants, by intervention group^1^

Characteristic	Intervention	Control	*P* value
	*n *= 74	*n *= 68	
*Child*			
Sex, male	37 (50)	36 (52)	0.66
Age, mo	13.0 ± 6.4	11.7 ± 6.2	0.25
Age at baseline, mo	13.0 ± 6.4	11.7 ± 6.2	0.25
Length-for-age, *z*-score	−1.1 ± 1.1	−0.7 ± 1.4	0.09
Weight-for-age, *z*-score	−0.8 ± 1.0	−0.8 ± 1.4	0.85
Weight-for-length, *z*-score	−0.3 ± 1.1	−0.4 ± 1.4	0.58
Minimum dietary diversity^2^	57	43	0.21
*Maternal*			
Age, y	27.4 ± 8.0	28.3 ± 7.4	0.49
BMI, kg/m^2^	22.6 ± 4.4	23.0 ± 4.2	0.56
Education completed			
None	8 (11)	13 (19)	0.28
Primary	33 (45)	24 (35)	
Secondary and higher	32 (44)	32 (46)	
Ethnicity^3^			
Krobo	65 (88)	39 (57)	<0.0001
Others	9 (12)	30 (43)	
Marital status			
Married/cohabiting	32 (49)	61 (88)	<0.0001
Not married	33 (51)	8 (12)	
Primary occupation			
Farmer	31 (51)	29 (45)	0.6
Trader/artisan	31 (51)	35 (55)	
Hygiene index^4^^,^^5^	0.01 ± 0.08	0.02 ± 0.11	0.54
Depressive symptoms score^6^	5.9 ± 4.5	7.2 ± 5.2	0.13
HOME score^4^^,^^7^	20.4 ± 4.9	19.2 ± 3.8	0.1
*Household*			
Assets index^8^	−0.29 ± 1.71	0.60 ± 2.07	0.007
Household size, #	7.1 ± 3.3	6.3 ± 2.4	0.09

1 Values are mean ± SD or *n* (%). Independent Student *t*-test for continuous variables and Pearson goodness-of-fit χ^2^ test for categorical variables. Intervention sample for age, education, marital status, occupation, hygiene index, and assets index ranged from 74 to 62. Control sample for length-for-age, weight-for-length, age, occupation, hygiene index, and assets index ranged from 68 to 64.

2 Children fed ≥4 of 7 food groups: grains, roots, and tubers; legumes and nuts; dairy products (milk, yogurt, and cheese); flesh food (meat, fish, poultry, and organ meats); eggs; vitamin A–rich foods and vegetables; and other fruits and vegetables.

3 Krobo: the local ethnic group; others: Akan, Ewe, Ga, among others.

4 Mother-child pair observations occurred 9 mo after the baseline measurements.

5 Hygiene index: fraction of the number of times mother washed her hands with soap and water before meal preparation, eating, feeding the child, and cleaning the child after defecation in the 6-h observation period.

6 Depressive symptoms scores: measured using the WHO Self-Reporting Questionnaire-20 (16).

7 The sum of positive responses out of a 45-item interview and observation that measured the amount and quality of stimulation and support provided to a child in the household during the 6-h observation period (15).

8 Assets index: the first component of a principal component analysis using 13 household assets: floor material, wall material, cooking fuel, electricity, and ownership of a telephone, radio, television, video player, DVD/CD player, refrigerator, sewing machine, motorcycle, and car.

### Description of child care received by maternal participation in NL

The most frequent type of care provided to NL-I and NL-C children was maternal supervision (52% compared with 48%; *P* < 0.01), followed by maternal direct care (32% compared with 34%; *P* = 0.04) (**Table 2**). Allocare was used in >10% of observation events whereas no child supervision was uncommon (4%).

**TABLE 2:** Distribution of child care observations, by intervention group^1^

Child care type	Intervention (*n *= 4465)	Control (*n *= 4451)	*P* value
Maternal direct care^2^	1411 (32)	1496 (34)	<0.04
Maternal supervisory care^3^	2335 (52)	2117 (48)	<0.01
Allocare^4^	552 (12)	649 (14)	<0.01
No supervision^5^	167 (4)	189 (4)	<0.22

1 Values are child care observations, *n* (%), in the 6-h observation period; *n *= total number of child care observations; *P* values generated from Pearson goodness-of-fit χ^2^ tests.

2 Mother directly engaged with child: bathing, cleaning, feeding, playing, talking to the child.

3 Mother not directly providing care but can see or hear the child.

4 Other persons provide child care in the presence or absence of the mother.

5 Child is without any supervision by mother or other person.

### Associations between child care, maternal project participation, and sociodemographic characteristics

Participation in the NL trial was not associated with maternal direct care, maternal supervisory care, any child care, and no supervision (**Table 3**). However, children of mothers in the NL-I group had 1.4-fold higher odds of allocare compared with those in the NL-C group. Child age was associated with any child care; compared with older children (>25 mo), younger children (11–17 mo and 18–23 mo) had a 2- and 4-fold higher odds of any child care, respectively. Children of mothers with no education compared with secondary education and higher had almost 6 times higher odds of any child care. Mothers who identified as currently working compared with those not currently working were almost twice more likely to leave their children unsupervised. Low maternal depressive symptoms were associated with 1.25-fold higher odds of maternal direct care, 12% lower odds of maternal supervisory care, and 20% lower odds of allocare. The children from households with low household assets had 1.25-fold higher odds of maternal direct care and 13% lower odds of maternal supervisory care.

**TABLE 3:** Associations between child care and maternal participation in the intervention arm of Nutrition Links and demographic characteristics^1^

	Maternal direct care	Maternal supervisory care	Allocare	No supervision	Any child care
*Group assignment*					
NL-I	1.07 (0.89, 1.28)	0.93 (0.78, 1.09)	1.36 (1.04, 1.79)*	0.64 (0.38, 1.10)	1.56 (0.91, 2.67)
NL-C (ref)					
*Child*					
Age,^2^ mo					
11–17	0.99 (0.85, 1.15)	1.08 (0.94, 1.24)	1.22 (0.98, 1.52)	0.47 (0.32, 0.68)***	2.14 (1.46, 3.12)***
18–23	1.19 (1.05, 1.36)**	1.01 (0.90, 1.15)	1.19 (0.98, 1.45) **	0.22 (0.16, 0.33) ***	4.46 (3.08, 6.46) ***
>25 (ref)					
*Maternal*					
Age, y					
<21	0.76 (0.65, 0.89)**	0.81 (0.70, 0.94)**	2.45 (1.95, 3.15)	1.37 (0.86, 2.17)	0.73 (0.46, 1.16)
21–34	1.09 (0.95, 1.27)	0.83 (0.72, 0.95)**	1.24 (0.98, 1.58)**	1.32 (0.86, 2.02)	0.75 (0.49, 1.16)
>35 (ref)					
Education					
No education	1.59 (1.32, 1.91)***	0.94 (0.79, 1.12)	0.68 (0.51, 0.90)**	0.17 (0.08, 0.36)***	5.92 (2.76, 12.71)***
Primary	1.20 (1.06, 1.36)**	1.21 (1.08, 1.37)**	0.53 (0.44, 0.64)***	0.76 (0.54, 1.07)	1.32 (0.94, 1.87)
Secondary and higher (ref)					
Currently working					
Yes	0.88 (0.78, 0.99)*	1.13 (1.01, 1.26)*	0.72 (0.61, 0.86)**	1.83 (1.36, 2.44)***	0.55 (0.41, 1.87)
No (ref)					
BMI, kg/m^2^					
<18.5	0.86 (0.72, 1.03)	1.34 (1.13, 1.58)**	0.79 (0.61, 1.03)	0.79 (0.47, 1.31)	1.27 (0.77, 2.11)
18.5–24.9	0.83 (0.72, 0.96)*	1.13 (0.99, 1.29)	1.13 (0.92, 1.38)	1.09 (0.75, 1.59)	0.92 (0.63, 1.34)
>25 (ref)					
Depressive symptoms^3^					
SRQ score <5	1.28 (1.14, 1.43)***	0.88 (0.79, 0.99)*	0.8 (0.67, 0.96)*	0.90 (0.64, 1.25)	1.12 (0.80, 1.56)
SRQ score ≥5 (ref)					
*Household*					
Assets index^4^					
Low	1.25 (1.10, 1.42)**	0.87 (0.77, 0.98)*	0.88 (0.74, 1.10)	0.99 (0.68, 1.27)	1.08 (0.79, 1.47)
High (ref)					
Household size (no.)					
<6	1.01 (0.89, 1.14)	0.87 (0.78, 0.97)*	1.27 (1.07, 1.51)**	1.03 (0.73, 1.44)	0.97(0.69, 1.37)
≥6					
Generalized χ^2^/Df	6.86	9.65	11.15	8.03	8.03
Sample size	120	120	120	120	120

1 Values are ORs (95% CI) compared with reference group; ^*,**,***^denotes significantly different: *P < 0.05; **P < 0.01; ***P < 0.001; generalized linear mixed models. PROC GLIMMIX accounted for clustering. NL-C, Nutrition Links control; NL-I, Nutrition Links intervention.

2 Age of children during mother-child pair observations.

3 Assessed using the SRQ-20: WHO Self-Reporting Questionnaire-20 (16).

4 Assets index: the first component of a principal component analysis using 13 household assets: floor material, wall material, cooking fuel, electricity, and ownership of a telephone, radio, television, video player, DVD/CD player, refrigerator, sewing machine, motorcycle, and car. Low = PCA assets index below the median; High = PCA assets index above the median.

## Discussion

This study assessed how participation in an NSA intervention affected mothers' time allocation to child care. The observation of women-child pairs participating in the intervention and control arms of the NL project did not reveal any differentials in the mothers’ time for care. In summary, this study found that participating in an NSA intervention was not associated with mothers' time for child care or any care received by the child. However, the odds of care provided by another person was associated with being part of the NL-I group.

Only a few agricultural intervention studies have assessed mothers' time allocated to child care. A review of mothers' employment in agriculture in India and time use found that agriculture did not always reduce total time for child care if there are other suitable caregivers, consistent with the results from the present study (23). A more recent study in Latin America showed that compared with employed mothers, those who were self-employed had more flexibility with their time and dedicated more time to child care (24). Self-employed mothers dedicated between 1.1 and 1.4 h more per week to child care in 4 Latin American countries. This suggests that women who have more control over their work, such as mothers participating in the NL-I, could be able to navigate constraints with time for child care better than mothers with formal employment. Women living in rural areas in Ghana and not in formal employment usually carry their infants and young children on their backs wherever they go. In addition, because of the communal way of living in rural communities, there are always a number of household members available to serve as allocare givers.

In our study, children of mothers with no education received more care compared with mothers with at least secondary education. Our results seem inconsistent with the existing evidence, mostly from high-income settings, that maternal education level is associated with time spent in child care. Two analyses using time-use surveys showed that college-educated mothers in the United States spent more time in child care compared with those with a high-school diploma, although this child gap has been shown to have narrowed in more recent surveys (25, 26). It would be helpful for future studies in rural agricultural contexts to further investigate how education affects child care. It is also important to note that more time spent on care does not necessarily translate to higher quality care, and this will be explored in future analysis.

Our current study showed that mothers who reported fewer depressive symptoms provided more care to their children. Our results are consistent with other studies showing negative associations between depressive symptoms in mothers and child nutrition, health, and development outcomes. A mother with depressed symptoms is less likely to feed her child a healthy diet, feed responsively, engage in learning activity and play with her child, and seek health care for the child (27, 28). A study in rural Zimbabwe further demonstrated the importance of maternal mental health for child health outcomes (29). The study found that a depressed mother was >30% less likely to have an institutional delivery and a fully immunized child. An important question for consideration is whether mothers from rural households who have elevated symptoms of depression should be recruited into NSA interventions that place additional demands on them. One might argue that the group interactions and social activities that are part of such integrated intervention could be beneficial because support for mental health in low- and middle-income settings is largely nonexistent. It would be helpful for programmers and researchers to measure depressive symptoms at baseline and other timepoints during nutrition-sensitive interventions and ensure that women with elevated symptoms are provided with the resources and support they need. In settings where there is very little to no support for mental health, the data collected on depressive symptoms can potentially become a tool for advocacy and change. In summary, maternal mental health should be given significant weight when investigating the effect of agricultural interventions on mothers' well-being and its link to child care and nutrition outcomes.

Contrary to our current results, some studies have found that participation in NSA interventions affected time for child care. In Nepal, participation in a vegetable, fruit, and cash crop program affected the time for care of children aged <5 y (30). The results were presented separately for male- and female-headed households. In female-headed households with a child aged <5 y, participating in the program was associated with 77 min less child care in a 12-h day compared with nonparticipants. In Bangladesh, a dairy value chain project that provided technical support with milk collection systems and improved access to high-quality inputs and services, compared time use of household members in participating compared with nonparticipating households (31). The study found that household time for child care (measured as weekly average hours dedicated to care) among participating households was 1 h less (β = −1.694; *P* < 0.05) relative to communities close in proximity to the program area, but interestingly almost 2 h more (β = 1.93; *P* < 0.05) relative to communities far removed from program areas. Differences in the comparison communities might explain this. The study also found that adult women from participating households spent 3 h more on dairy-related activities than communities far removed from the program areas, lending more weight to the program's impact on child care. Neither of the studies above randomly assigned participants to the program arm and could have been at risk of selection bias. Compared with homestead agriculture interventions like the NL trial, which involved small livestock and was situated in the home, interventions that require a higher intensity of work (large livestock) and that happen outside the home are likely to affect child care differently. In summary, NSA interventions that seek to ensure that mothers are not overburdened must consider the location of the work and its intensity as important variables.

Our study has a number of strengths and limitations. The first strength is that we used in-home observation of mother-child pairs, which is considered the gold standard of time-use measurements. The examination of all categories of mothers' time use across the 6-h observation period and our ability to account for allocare was another strength of this study. Our study is one of the few that have investigated maternal time use within the context of an NSA intervention and adds important evidence to the available literature. Yet, there are some limitations. Our observations lasted only 6 h for each mother-child pair. A longer observation period might have resulted in different distributions of time use. The presence of 2 observers in the household might also have altered women's normal routines, although extreme care was taken to assure that the women carried out their routine activities as a normal day and did not know exactly when the research team would arrive in the household. Also, the data collection period coincided with the dry agricultural season (November to December), which is likely to be associated with fewer agricultural activities in most households. Observations during the major agricultural season might have resulted in different time allocations patterns. Finally, due to missing covariates, ∼10% of the sample were lost after running our models. This presents a potential source of bias.

Our results are indirectly corroborated by 2 studies that were carried out as part of the NL project, including this subset of women. The first, an intention-to-treat analysis showed that the NL intervention improved child's length-for-age/height-for-age *z*-scores (β = 0.22; 95% CI: 0.09, 0.34) and minimum dietary diversity [adjusted odds ratio (aOR) = 1.65; 95% CI: 1.02, 2.69] (10). The second, a sensitivity analysis that stratified the NL-I women by participation level in the intervention (high, medium, or low) found that compared with the NL-C group, the high-participation NL-I group was associated with higher egg consumption (aOR = 2.87; 95% CI: 1.20, 6.85) and height-for-age *z*-scores (β = 0.45; 95% CI: 0.17, 0.73) (32). There is, therefore, evidence that maternal participation did not negatively affect child nutrition. Therefore, this current analysis confirms that mothers in the NL-I navigated their time constraints with child care successfully.

Measuring time use in rural agricultural communities with poor roads and infrastructure is labor- and time-intensive, and these factors contribute to the limited research on the topic. Efforts should be invested in standardizing new, quick, and easy-to-use measurement tools to guide future studies and build high-quality evidence on maternal time use in low- and middle-income countries. Time-use research would also greatly benefit from a mixed-methods approach. An in-depth qualitative analysis of the aspects of time allocation that women find challenging would have provided nuanced data to support our results and should be considered in future studies to guide strategies to prevent potential unintended consequences of program participation on women's time for care.

## Supplementary Material

nzac145_Supplemental_Files

## Data Availability

The data described in this manuscript and SAS analytic codes will be made available upon a formal request and approval by the principal investigator (GSM).

## Notes

The Government of Canada provided funding for this initiative through a grant from Global Affairs Canada (Grant no. S065653). World Vision Canada provided logistical support for fieldwork. Heifer International provided poultry and technical training to women in the intervention arm. None of these nongovernmental funding entities were involved in the design, analysis, and interpretation of the data.

Author disclosures: The authors report no conflicts of interest. GSM is an Associate Editor for *Advances in Nutrition* and the series’ Deputy Editor for *Current Developments in Nutrition* and played no role in the Journal's evaluation of the manuscript.

Supplemental Appendices A and B are available from the “Supplementary data” link in the online posting of the article and from the same link in the online table of contents at https://academic.oup.com/cdn/.

Abbreviations used: aOR, adjusted odds ratio; HOME, Home Observation for Measurement of the Environment; NL, Nutrition Links; NL-C, Nutrition Links control group; NL-I, Nutrition Links intervention group; NSA, nutrition-sensitive agriculture; SRQ-20, 20-item Self Reporting Questionnaire; UMK, Upper Manya Krobo.
