# Supplementary material for: Participating in a Nutrition-Sensitive Agriculture Intervention Is Not Associated with Less Maternal Time for Care in a Rural Ghanaian District
Source: Curr Dev Nutr. 2022 Sep 29;6(10):nzac145. doi: 10.1093/cdn/nzac145 (PMC9718649; doi:10.1093/cdn/nzac145)
Supplement: nzac145_Supplemental_Files [file nzac145_supplemental_files.zip › Appendix A_Mothers time allocation to child care form.docx]

1. **VISIT INFORMATION**
   1. **Interviewer’s name and code**________________________________ ID#.....................................................................................................|__|__|__|
   2. **Date of visit** (dd/mm/yy*)…………………………………………*………………………. …………………………………………………|__|__||__|__| 20|__|__|
   3. **Was visit missed?..................................................................................................................................................................................................** |__|__|

**0- No 1-Yes**

- 1. **If missed why and what is the rescheduled date agreed on? ___________________________________________________________________**

**_____________________________________________________________________________________________________**|__|__||__|__| 20|__|__|

- 1. **Time of Arrival at household………………………………………………………………………………………………………………………...**|__|__| : |__|__|
  2. **Start time of 6hr- observation……………………………………………………………………………………………………………………….**|__|__| : |__|__|
  3. **End time of 6hr-observation…………………………………………………………………………………………………………………………**|__|__| : |__|__|

1. **Preliminary Questions**
   1. **Has anything happened since we arranged the meeting to make this 6-hr observation impossible today?........................................** |__|__|

**1. Yes 2. No (If no skip 2.2)**

- 1. **If yes, please can you tell us what it is? .....................................................................................................................................................** |__|__|

**1. Someone died (funeral) 2. Illness of mother or child 3. Other 4. If Other specify-----------------------------------------------------------------------------**

- 1. **Is today a typical day for you?.........................................................................................................................................................................** |__|__|

**1. Yes 2. No**

- 1. **If no, what is unusual about today?...............................................................................................................................................................** |__|__|

1. **Festival**
2. **Harvesting period**
3. **Other 3.1 Other Specify______________________________**
4. **Not applicable**
   1. **Will this activity change the usual activities you do daily ...........................................................................................................................** |__|__|

**1. Yes 2. No**

- 1. **If yes, how will it change the usual activities you do daily? _________________________________________________________________**

**____________________________________________________________________________________________________________________**

**____________________________________________________________________________________________________________________**

- 1. **Will you leave {Name} [ index child] with other people (caretakers) the entire day because of this ? .....................................................** |__|__|

1. **Yes. 2. No**
   1. **If yes, who will you leave your child with? .....................................................................................................................................................** |__|__|
2. **Grandmother (elderly relative) 2. Other relative 3 Older sibling (15 years and above) 4. Older sibling (less than 15 years) 5. Father 6. Neighbour**

**3.0 6-HR OBSERVATION**

***Please read the following instructions before you start the 6-hr observation:***

***Explain to the mother that you are interested in how she spends her day and tell her not to change her usual activities because of your presence. Remind her not to worry about food or water for you and that you came prepared. All activities that the mother does alone or with INDEX child and other family members must be reported on this form.***

***Script:***

***P****lease we will start observing you from now. If for some reason, we cannot see what you are doing we will ask you to tell us. Other people (family friend and neighbours) who may help you with your activities will also be observed but we will ask if this is ok with them before we do so*

| **Time of observation** | | **Time Code** | **What is the Mother doing? *Write down all the tasks the mother is doing at the same time (each activity should be on a separate line) Please specify whether the activity is being done with the index child or with other children***  ***Examples;***  *Talking to child; listening to child; picking up child; putting down child; carrying child in arms; carrying child on back; watching child, sitting with child; lying down with child; feeding/bathing /playing with child.*  *Tending to garden; tending to animals; household cleaning; laundry; food preparation.*  *Eating; bathing; talking with other family member; talking with neighbour/friend; leisure.* | **Can the mother hear or see the index child?**  **1.Yes**  **2. No** | **Activity code** | **Place of Activity**   1. **Home compound** 2. **Farm** 3. **Market** 4. **Travel** 5. **Other-Specify** | **Persons together at the time of activity**   1. **Mother** 2. **index child** 3. **Spouse** 4. **Elderly relatives** 5. **Other relatives** 6. **Neighbours** 7. **With child(ren)-including index child)** 8. **With child(ren)-excluding the index child)** 9. **other (Please specify)**   **Elderly- grandmother, grandfather , grand aunts and uncles**  **Together- They can all see each other** |
| --- | --- | --- | --- | --- | --- | --- | --- |
|  | |  |  |  |  |  |  |
| 6:00 | | 6 | feeding child |  | C03 | 1 | 1,2,7 |
| 6:00 | | 7 | talking to child |  | C07 | 1 | 1,2,7 |
| **From** | **To** | **Time Code** | **What is the Mother doing?** | **See or hear child**  **1.Yes**  **2. No** | **Activity code** | **Place of Activity** | **Persons together at the time of activity** |
|  |  |  |  |  |  |  |  |
|  |  |  |  |  |  |  |  |
|  |  |  |  |  |  |  |  |
|  |  |  |  |  |  |  |  |
|  |  |  |  |  |  |  |  |
|  |  |  |  |  |  |  |  |
|  |  |  |  |  |  |  |  |
|  |  |  |  |  |  |  |  |
|  |  |  |  |  |  |  |  |
| **From** | **To** | **Time Code** | **What is the Mother doing?** | **See or hear child**  **1.Yes**  **2. No** | **Activity code** | **Place of Activity** | **Persons together at the time of activity** |
|  |  |  |  |  |  |  |  |
|  |  |  |  |  |  |  |  |
|  |  |  |  |  |  |  |  |
|  |  |  |  |  |  |  |  |
|  |  |  |  |  |  |  |  |
|  |  |  |  |  |  |  |  |
|  |  |  |  |  |  |  |  |
|  |  |  |  |  |  |  |  |
|  |  |  |  |  |  |  |  |
| **From** | **To** | **Time Code** | **What is the Mother doing?** | **See or hear child**  **1.Yes**  **2. No** | **Activity code** | **Place of Activity** | **Persons together at the time of activity** |
|  |  |  |  |  |  |  |  |
|  |  |  |  |  |  |  |  |
|  |  |  |  |  |  |  |  |
|  |  |  |  |  |  |  |  |
|  |  |  |  |  |  |  |  |
|  |  |  |  |  |  |  |  |
|  |  |  |  |  |  |  |  |
|  |  |  |  |  |  |  |  |
|  |  |  |  |  |  |  |  |
| **From** | **To** | **Time Code** | **What is the Mother doing?** | **See or hear child**  **1.Yes**  **2. No** | **Activity code** | **Place of Activity** | **Persons together at the time of activity** |
|  |  |  |  |  |  |  |  |
|  |  |  |  |  |  |  |  |
|  |  |  |  |  |  |  |  |
|  |  |  |  |  |  |  |  |
|  |  |  |  |  |  |  |  |
|  |  |  |  |  |  |  |  |
|  |  |  |  |  |  |  |  |
|  |  |  |  |  |  |  |  |
|  |  |  |  |  |  |  |  |
| **From** | **To** | **Time Code** | **What is the Mother doing?** | **See or hear child**  **1.Yes**  **2. No** | **Activity code** | **Place of Activity** | **Persons together at the time of activity** |
|  |  |  |  |  |  |  |  |
|  |  |  |  |  |  |  |  |
|  |  |  |  |  |  |  |  |
|  |  |  |  |  |  |  |  |
|  |  |  |  |  |  |  |  |
|  |  |  |  |  |  |  |  |
|  |  |  |  |  |  |  |  |
|  |  |  |  |  |  |  |  |
|  |  |  |  |  |  |  |  |
| **From** | **To** | **Time Code** | **What is the Mother doing?** | **See or hear child**  **1.Yes**  **2. No** | **Activity code** | **Place of Activity** | **Persons together at the time of activity** |
|  |  |  |  |  |  |  |  |
|  |  |  |  |  |  |  |  |
|  |  |  |  |  |  |  |  |
|  |  |  |  |  |  |  |  |
|  |  |  |  |  |  |  |  |
|  |  |  |  |  |  |  |  |
|  |  |  |  |  |  |  |  |
|  |  |  |  |  |  |  |  |
|  |  |  |  |  |  |  |  |
| **From** | **To** | **Time Code** | **What is the Mother doing?** | **See or hear child**  **1.Yes**  **2. No** | **Activity code** | **Place of Activity** | **Persons together at the time of activity** |
|  |  |  |  |  |  |  |  |
|  |  |  |  |  |  |  |  |
|  |  |  |  |  |  |  |  |
|  |  |  |  |  |  |  |  |
|  |  |  |  |  |  |  |  |
|  |  |  |  |  |  |  |  |
|  |  |  |  |  |  |  |  |
|  |  |  |  |  |  |  |  |
|  |  |  |  |  |  |  |  |
| **From** | **To** | **Time Code** | **What is the Mother doing?** | **See or hear child**  **1.Yes**  **2. No** | **Activity code** | **Place of Activity** | **Persons together at the time of activity** |
|  |  |  |  |  |  |  |  |
|  |  |  |  |  |  |  |  |
|  |  |  |  |  |  |  |  |
|  |  |  |  |  |  |  |  |
|  |  |  |  |  |  |  |  |
|  |  |  |  |  |  |  |  |
|  |  |  |  |  |  |  |  |
|  |  |  |  |  |  |  |  |
|  |  |  |  |  |  |  |  |
| **From** | **To** | **Time Code** | **What is the Mother doing?** | **See or hear child**  **1.Yes**  **2. No** | **Activity code** | **Place of Activity** | **Persons together at the time of activity** |
|  |  |  |  |  |  |  |  |
|  |  |  |  |  |  |  |  |
|  |  |  |  |  |  |  |  |
|  |  |  |  |  |  |  |  |
|  |  |  |  |  |  |  |  |
|  |  |  |  |  |  |  |  |
|  |  |  |  |  |  |  |  |
|  |  |  |  |  |  |  |  |
|  |  |  |  |  |  |  |  |
| **From** | **To** | **Time Code** | **What is the Mother doing?** | **See or hear child**  **1.Yes**  **2. No** | **Activity code** | **Place of Activity** | **Persons together at the time of activity** |
|  |  |  |  |  |  |  |  |
|  |  |  |  |  |  |  |  |
|  |  |  |  |  |  |  |  |
|  |  |  |  |  |  |  |  |
|  |  |  |  |  |  |  |  |
|  |  |  |  |  |  |  |  |
|  |  |  |  |  |  |  |  |
|  |  |  |  |  |  |  |  |
|  |  |  |  |  |  |  |  |
| **From** | **To** | **Time Code** | **What is the Mother doing?** | **See or hear child**  **1.Yes**  **2. No** | **Activity code** | **Place of Activity** | **Persons together at the time of activity** |
|  |  |  |  |  |  |  |  |
|  |  |  |  |  |  |  |  |
|  |  |  |  |  |  |  |  |
|  |  |  |  |  |  |  |  |
|  |  |  |  |  |  |  |  |
|  |  |  |  |  |  |  |  |
|  |  |  |  |  |  |  |  |
|  |  |  |  |  |  |  |  |
|  |  |  |  |  |  |  |  |
| **From** | **To** | **Time Code** | **What is the Mother doing?** | **See or hear child**  **1.Yes**  **2. No** | **Activity code** | **Place of Activity** | **Persons together at the time of activity** |
|  |  |  |  |  |  |  |  |
|  |  |  |  |  |  |  |  |
|  |  |  |  |  |  |  |  |
|  |  |  |  |  |  |  |  |
|  |  |  |  |  |  |  |  |
|  |  |  |  |  |  |  |  |
|  |  |  |  |  |  |  |  |
|  |  |  |  |  |  |  |  |
|  |  |  |  |  |  |  |  |
| **From** | **To** | **Time Code** | **What is the Mother doing?** | **See or hear child**  **1.Yes**  **2. No** | **Activity code** | **Place of Activity** | **Persons together at the time of activity** |
|  |  |  |  |  |  |  |  |
|  |  |  |  |  |  |  |  |
|  |  |  |  |  |  |  |  |
|  |  |  |  |  |  |  |  |
|  |  |  |  |  |  |  |  |
|  |  |  |  |  |  |  |  |
|  |  |  |  |  |  |  |  |
|  |  |  |  |  |  |  |  |
|  |  |  |  |  |  |  |  |
| **From** | **To** | **Time Code** | **What is the Mother doing?** | **See or hear child**  **1.Yes**  **2. No** | **Activity code** | **Place of Activity** | **Persons together at the time of activity** |
|  |  |  |  |  |  |  |  |
|  |  |  |  |  |  |  |  |
|  |  |  |  |  |  |  |  |
|  |  |  |  |  |  |  |  |
|  |  |  |  |  |  |  |  |
|  |  |  |  |  |  |  |  |
|  |  |  |  |  |  |  |  |
|  |  |  |  |  |  |  |  |
|  |  |  |  |  |  |  |  |

**4.0 6hr- Observation Summary Questions**

**4.1 Will you say today has been a typical day so far?**

1. **Yes 0. No...........................................................................................................................................................................................................**|__|__|

**4.1.1 If not, what was unusual about your so far?**

**………………………………………………………………………………………………………………………………………………………………………………………………………………………**

**…………………………………………………………………………………………………………………………………………………………………………………………………………………………**

**……………………………………………………………………………………………………………………………………………………………………………………………………………………....**

**CODES**

| **Income related activities** | **Nlinks associated work** | **Child care**  **Activities /Personal care** | **House chores** | **Sleep/Leisure and social activities** |
| --- | --- | --- | --- | --- |
| Selling goods at a stationary place  **E01** | Feeding chickens  **N01** | Preparing food for child  **C01** | Preparing family meals  **H01** | Sleep/napping  **L01** |
| Hawking goods  **E02** | Water for chickens  **N02** | Breastfeeding  **C02** | Cleaning (sweeping,  scrubbing)  **H02** | Chatting with family/neighbours  **L02** |
| Weeding field  **E03** | Cleaning coop  **N03** | Feeding complementary foods  **C03** | Collecting water  **H03** | Attending church  **L03** |
| Planting crops  **E04** | Repairing coop  **N04** | Bathing child  **C04** | Collecting firewood  **H03** | Attending community meetings (civil responsibilities)  **L04** |
| Harvesting crops  **E05** | Collecting eggs  **N05** | Cleaning child after defecation  **C05** | Processing food (pounding, grinding etc)  **H04** | Listening to radio  **L05** |
| Other income related activity  **E06** | Attending to garden.  **N06** | Playing with child  **C06** | Buying foodstuffs from market  **H05** | Watching t.v  **L06** |
|  | Nutrition education sessions  **N07** | talking with child  **C07** | Washing Clothes  **H06** | Other leisure/social activities specify these  **L07** |
|  | POG egg collection activities  **N08** | Taking child to child welfare clinic  **C08** | Other house related work specify  **H07** |  |
|  | Other Nutrition Links associated work specify…  **N09** | Taking sick child to hospital/CHPS  **C09** |  |  |
|  |  | Mother’s self-hygiene  (bathing /self-grooming)  **C10** |  |  |
|  |  | Mother eating  **C11** |  |  |
