# Supplementary material for: Participating in a Nutrition-Sensitive Agriculture Intervention Is Not Associated with Less Maternal Time for Care in a Rural Ghanaian District
Source: Curr Dev Nutr. 2022 Sep 29;6(10):nzac145. doi: 10.1093/cdn/nzac145 (PMC9718649; doi:10.1093/cdn/nzac145)
Supplement: nzac145_Supplemental_Files [file nzac145_supplemental_files.zip › Appendix B_Index child observation form.docx]

1. **VISIT INFORMATION**
   1. **Interviewer’s name and code**________________________________ ID#.....................................................................................................|__|__|__|
   2. **Date of visit** (dd/mm/yy*) …………………………………………*………………………. …………………………………………………|__|__||__|__| 20|__|__|
   3. **Write down the start time for observing index child………………………………………………………………………..........**|__|__| : |__|__|:::|__|__|__|
   4. **Write down the end time for observing index child…………………………………………...................................................**|__|__| : |__|__|:::|__|__|__|

*Please read the following instructions before you start the 6-hr observation:*

***This form is solely for observing the index child. The mother may have two infants under the age of five and so please make sure you know exactly who the index child is. As soon as you have correctly verified the identity of the index child record all activities involving the child on this form.***

| **Time of Activity** | | **Time Code** | **What is being done with/ to the index child?**  **Example;**  child is eating, child is being cleaned/bathed/ child is playing etc | **Who is engaged in current activity with index child**   1. **Mother** 2. **Spouse** 3. **Sibling-specify age** 4. **Elderly relatives** 5. **Other relatives** 6. **Neighbours** 7. **With child(ren)-including index child)** 8. **other (Please specify)** | **Place of activity**   1. **Home compound** 2. **Farm** 3. **Market** 4. **Travel** 5. **Other-Specify** | **Persons together at the time of activity**   1. **Mother** 2. **index child** 3. **Spouse** 4. **Elderly relatives** 5. **Other relatives** 6. **Neighbours** 7. **With child(ren)-including index child)** 8. **With child(ren)-excluding the index child)** 9. **other (Please specify** |
| --- | --- | --- | --- | --- | --- | --- |
| **From** | **To** |  |  |  |  |  |
|  |  |  |  |  |  |  |
|  |  |  |  |  |  |  |
|  |  |  |  |  |  |  |
|  |  |  |  |  |  |  |
| **From** | **To** | **Time Code** | **What is being done with/ to the index child?** | **Who is engaged in current activity with index child** | **Place of activity** | **Persons together at the time of activity** |
|  |  |  |  |  |  |  |
|  |  |  |  |  |  |  |
|  |  |  |  |  |  |  |
|  |  |  |  |  |  |  |
|  |  |  |  |  |  |  |
|  |  |  |  |  |  |  |
|  |  |  |  |  |  |  |
|  |  |  |  |  |  |  |
| **From** | **To** | **Time Code** | **What is being done with/ to the index child?** | **Who is engaged in current activity with index child** | **Place of activity** | **Persons together at the time of activity** |
|  |  |  |  |  |  |  |
|  |  |  |  |  |  |  |
|  |  |  |  |  |  |  |
|  |  |  |  |  |  |  |
|  |  |  |  |  |  |  |
|  |  |  |  |  |  |  |
|  |  |  |  |  |  |  |
|  |  |  |  |  |  |  |
| **From** | **To** | **Time Code** | **What is being done with/ to the index child?** | **Who is engaged in current activity with index child** | **Place of activity** | **Persons together at the time of activity** |
|  |  |  |  |  |  |  |
|  |  |  |  |  |  |  |
|  |  |  |  |  |  |  |
|  |  |  |  |  |  |  |
|  |  |  |  |  |  |  |
|  |  |  |  |  |  |  |
|  |  |  |  |  |  |  |
|  |  |  |  |  |  |  |
| **From** | **To** | **Time Code** | **What is being done with/ to the index child?** | **Who is engaged in current activity with index child** | **Place of activity** | **Persons together at the time of activity** |
|  |  |  |  |  |  |  |
|  |  |  |  |  |  |  |
|  |  |  |  |  |  |  |
|  |  |  |  |  |  |  |
|  |  |  |  |  |  |  |
|  |  |  |  |  |  |  |
|  |  |  |  |  |  |  |
|  |  |  |  |  |  |  |
| **From** | **To** | **Time Code** | **What is being done with/ to the index child?** | **Who is engaged in current activity with index child** | **Place of activity** | **Persons together at the time of activity** |
|  |  |  |  |  |  |  |
|  |  |  |  |  |  |  |
|  |  |  |  |  |  |  |
|  |  |  |  |  |  |  |
|  |  |  |  |  |  |  |
|  |  |  |  |  |  |  |
|  |  |  |  |  |  |  |
|  |  |  |  |  |  |  |
| **From** | **To** | **Time Code** | **What is being done with/ to the index child?** | **Who is engaged in current activity with index child** | **Place of activity** | **Persons together at the time of activity** |
|  |  |  |  |  |  |  |
|  |  |  |  |  |  |  |
|  |  |  |  |  |  |  |
|  |  |  |  |  |  |  |
|  |  |  |  |  |  |  |
|  |  |  |  |  |  |  |
|  |  |  |  |  |  |  |
|  |  |  |  |  |  |  |
| **From** | **To** | **Time Code** | **What is being done with/ to the index child?** | **Who is engaged in current activity with index child** | **Place of activity** | **Persons together at the time of activity** |
|  |  |  |  |  |  |  |
|  |  |  |  |  |  |  |
|  |  |  |  |  |  |  |
|  |  |  |  |  |  |  |
|  |  |  |  |  |  |  |
|  |  |  |  |  |  |  |
|  |  |  |  |  |  |  |
|  |  |  |  |  |  |  |
| **From** | **To** | **Time Code** | **What is being done with/ to the index child?** | **Who is engaged in current activity with index child** | **Place of activity** | **Persons together at the time of activity** |
|  |  |  |  |  |  |  |
|  |  |  |  |  |  |  |
|  |  |  |  |  |  |  |
|  |  |  |  |  |  |  |
|  |  |  |  |  |  |  |
|  |  |  |  |  |  |  |
|  |  |  |  |  |  |  |
|  |  |  |  |  |  |  |
| **From** | **To** | **Time Code** | **What is being done with/ to the index child?** | **Who is engaged in current activity with index child** | **Place of activity** | **Persons together at the time of activity** |
|  |  |  |  |  |  |  |
|  |  |  |  |  |  |  |
|  |  |  |  |  |  |  |
|  |  |  |  |  |  |  |
|  |  |  |  |  |  |  |
|  |  |  |  |  |  |  |
|  |  |  |  |  |  |  |
|  |  |  |  |  |  |  |
| **From** | **To** | **Time Code** | **What is being done with/ to the index child?** | **Who is engaged in current activity with index child** | **Place of activity** | **Persons together at the time of activity** |
|  |  |  |  |  |  |  |
|  |  |  |  |  |  |  |
|  |  |  |  |  |  |  |
|  |  |  |  |  |  |  |
|  |  |  |  |  |  |  |
|  |  |  |  |  |  |  |
|  |  |  |  |  |  |  |
|  |  |  |  |  |  |  |
| **From** | **To** | **Time Code** | **What is being done with/ to the index child?** | **Who is engaged in current activity with index child** | **Place of activity** | **Persons together at the time of activity** |
|  |  |  |  |  |  |  |
|  |  |  |  |  |  |  |
|  |  |  |  |  |  |  |
|  |  |  |  |  |  |  |
|  |  |  |  |  |  |  |
|  |  |  |  |  |  |  |
|  |  |  |  |  |  |  |
|  |  |  |  |  |  |  |
| **From** | **To** | **Time Code** | **What is being done with/ to the index child?** | **Who is engaged in current activity with index child** | **Place of activity** | **Persons together at the time of activity** |
|  |  |  |  |  |  |  |
|  |  |  |  |  |  |  |
|  |  |  |  |  |  |  |
|  |  |  |  |  |  |  |
|  |  |  |  |  |  |  |
|  |  |  |  |  |  |  |
|  |  |  |  |  |  |  |
|  |  |  |  |  |  |  |

**CHILD CARE CODES**

| **C01**  Breastfeeding  child | **C02**  Eating complementary foods | **C03**  Eating an  egg | **C04**  Child is being bathed | **C05**  Child is being cleaned after defecation | **C06**  Child is playing | **C07**  Child is being carried at the back | **C08**  Child is being taken to the CWC | **C09**  Child is being taken to the clinic | **C10**  Child is  playing |
| --- | --- | --- | --- | --- | --- | --- | --- | --- | --- |
